# Design, Rational Repurposing, Synthesis, In Vitro Evaluation, Homology Modeling and In Silico Study of Sulfuretin Analogs as Potential Antileishmanial Hit Compounds

**DOI:** 10.3390/ph15091058

**Published:** 2022-08-26

**Authors:** Ahmed H.E. Hassan, Trong-Nhat Phan, Yeonwoo Choi, Suyeon Moon, Joo Hwan No, Yong Sup Lee

**Affiliations:** 1 Department of Medicinal Chemistry, Faculty of Pharmacy, Mansoura University, Mansoura 35516, Egypt; 2 Medicinal Chemistry Laboratory, Department of Pharmacy, College of Pharmacy, Kyung Hee University, Seoul 02447, Korea; 3 Host-Parasite Research Laboratory, Institut Pasteur Korea, Seongnam-si 13488, Gyeonggi-do, Korea; 4 Department of Fundamental Pharmaceutical Sciences, Kyung Hee University, Seoul 02447, Korea; 5 Department of Life and Nanopharmaceutical Sciences, Kyung Hee University, Seoul 02447, Korea

**Keywords:** antileishmanial agents, repurposing, *Leishmania donovani*, promastigotes, fumarate reductase

## Abstract

Direct growth inhibition of infectious organisms coupled with immunomodulation to counteract the immunosuppressive environment might be a beneficial therapeutic approach. Herein, a library of sulfuretin analogs were developed with potential capabilities to inhibit production of the immunosuppressive PGE_2_ and elicit direct growth inhibition against *Leishmania donovani*; the major causative agent of the fatal visceral leishmaniasis. Amongst explored library members bearing diverse methoxy and/or hydroxy substitution patterns at rings B and A, analog **1i** retaining the C6-hydroxy moiety at ring-A, but possessing methoxy moieties in place of the polar dihydroxy moieties of sulfuretin ring-B, as well as analog **1q** retaining the sulfuretin′s polar dihydroxy moieties at ring-B, but incorporating a C6-methoxy moiety instead of the C6-hydroxy moiety at ring-A, were the most promising hit compounds. Cytotoxicity evaluation suggested that analog **1i** possesses a safety profile inducing the death of the parasite rather than host cells. In silico simulation provided insights into their possible binding with *Leishmania donovani* fumarate reductase. The current investigation presents sulfuretin analogs **1i** and **1q** as potential hit compounds for further development of multifunctional therapeutic agents against visceral leishmaniasis.

## 1. Introduction

Infectious diseases had gravely affected mankind since ancient history. Historical outbreaks of several infectious disease and their severe consequences are documented not only in the modern era, but also in ancient and medieval ages. To date, several infectious diseases still present serious threats despite the current progress in understanding the biology of pathogenic organisms and efforts to develop chemotherapeutic agents. Unfortunately, high attention was payed to infectious disease with pandemic potential, but some tropical infectious diseases have received little attention and were largely neglected. Leishmaniasis, one of the infectious neglected tropical diseases (NTDs), is caused by protozoa of genus *Leishmania* after bites from infected sandflies. Although the causative protozoan parasite of leishmaniasis was not described until it was discovered independently by William Boog Leishman in 1900 and Charles Donovan in 1903, fossil evidences showed ancient members of the genus *Leishmania* parasite in the alimentary tract of 100 million-year-old fossilized sandflies [1]. Despite being present since ancient prehistoric ages until today, currently available clinical treatments are not satisfactory in terms of toxicities or efficacy [2]. There is a need to develop new therapeutic agents, especially for visceral leishmaniasis, mainly caused by *Leishmania donovani*, which is the life-threating form out of the different leishmaniasis clinical forms and the second most mortal parasitic disease after malaria. 

Immune response dynamics are an important component in infectious diseases. Evasion from host defense mechanisms is vital for intracellular parasites, such as *leishmania*, to proliferate and spread. Although the causes for immune system failure to curb the growth and spread of visceral leishmaniasis are not well understood, outcomes of recent research unveiled prostaglandin E_2_ (PGE_2_) as an important player. Macrophage′s PGE_2_ production is upregulated by *L. donovani*, which negatively regulates immune response. Thus, it contributes to survival and systemic spread of the parasite [3,4,5]. Consequently, combating the *L. donovani*-induced immunosuppressed environment through inhibition of macrophages PGE_2_ production might be beneficial in visceral leishmaniasis treatment.

Fumarate reductase is an interesting target for developing therapeutic agents for several parasitic diseases [6]. Two fumarate reductases are known: quinol-dependent fumarate reductase and NADH-dependent fumarate reductase. Both reduce fumarate to succinate as a part of anaerobic respiration and energy production in several parasites. The NADH-dependent fumarate reductase has been found as an indispensable component of the respiratory chain of promastigotes and amastigotes of *L. donovani*, but not for mammalian cells [7,8]. Consequently, development of NADH-dependent fumarate reductase inhibitors would be an attractive approach to develop antileishmanial agents [9].

Targeting multiple pathways involved in a disease has been a successful strategy to achieve synergy, reduce side effects and minimize resistance [10,11]. While a combination therapy might be used, multifunctional molecules are better as they offer less molecularity, reduced side effects, and improved ADMET [12]. Considering these benefits, this effort aimed to develop multifunctional molecules against visceral leishmaniasis caused by *L. donovani*, which is the most lethal form of leishmaniasis. 

## 2. Results

### 2.1. Design and Repurposing Rational

**Figure 1:**
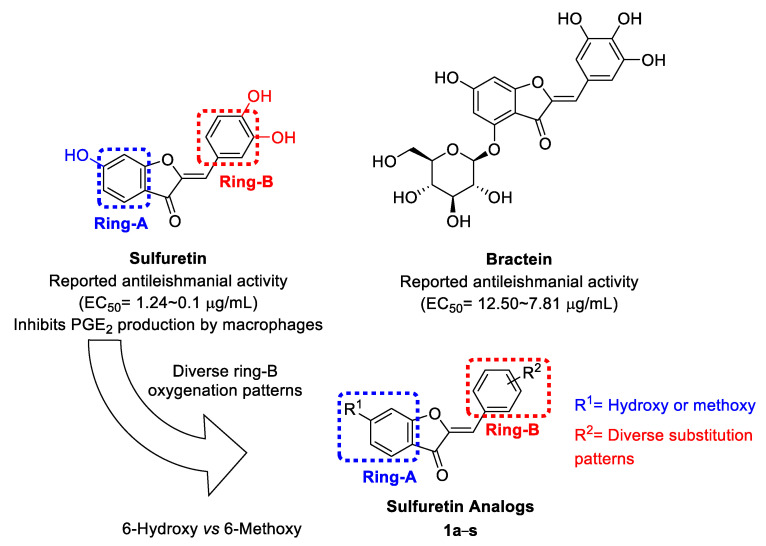
Natural aurones with reported antileishmanial activity and rational for development of antileishmanial sulfuretin analogs.

Natural products are a fascinating reservoir of starting hits and lead compounds, which proved to be successful starting points for further development [13,14]. Currently, natural products-based drug discovery and development is an important approach towards development of therapeutic agents. Sulfuretin (isolated from *Rhus verniciflua*, Figure 1) and bractein (isolated from *Helichrysum bracteatum*, Figure 1) are two interesting aurone-based natural products that have been reported to trigger antileishmanial activity [15]. Structurally, bractein differs from sulfuretin by incorporating a 4-*O*-glucoside moiety at ring-A and by the presence of an additional 5′-hydroxy moiety at ring-B. Both sulfuretin and bractein were found to inhibit NADH-dependent fumarate reductase of *L.* *major* [16]. However, the structural differences between bractein and sulfuretin resulted in a reduced antileishmanial activity for bractein relative to sulfuretin. To the best of our knowledge, information is scant regarding potential antileishmanial activity of sulfuretin analogs and aurones in general [17]. Structural variations to access sulfuretin analogs might afford better antileishmanial hit compounds.

Interestingly, a series of sulfuretin analogs is known to inhibit macrophage′s production of PGE_2_ [18]; an important player that triggers the immunosuppressed environment and promotes survival and systemic spread of the parasite in visceral leishmaniasis caused by *L. donovani*. Such inhibition of PGE_2_ production would result in immunomodulation that might restore immune response against *L. donovani*. If members of this series can elicit direct antileishmanial effects, then this class of molecules might be developed as multifunctional compounds, combining direct and indirect antileishmanial effects through inhibiting the viability of leishmania and preventing negative immunomodulation because of PGE_2_ production. As shown in Figure 1, a sulfuretin analogs library that included ring-B and/or ring-A modified analogs was intended to be explored. As oxygenation of natural products in the skeleton is common through hydroxy and/or methoxy substituents, members of the targeted library of sulfuretin analogs involved diverse hydroxy/methoxy oxygenation patterns at ring-B coupled with retaining sulfuretin′s C6-hydroxy moiety on ring-A or conversion into C6-methoxy moiety (Figure 1). Members of the explored library are enumerated in Table 1 alongside their known PGE_2_ inhibitory activities [18].

### 2.2. Synthesis of Sulfuretin Analogs 

**Scheme 1:**
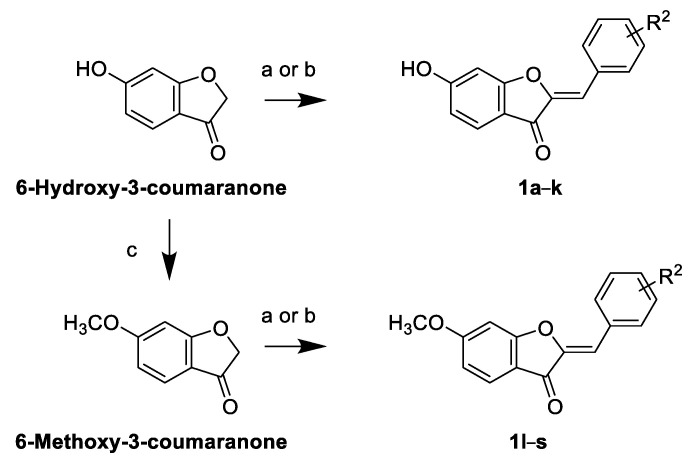
Reagents and reaction conditions: (**a**) 12 N HCl, ethanol, 60–70 °C, for the specified time; (**b**) 50% KOH, methanol, 60 °C, or the specified time; (**c**) Iodomethane, K_2_CO_3_, DMF, rt, overnight.

Concise synthesis was conducted to access the targeted sulfuretin analogs. Thus, analogs **1a**–**k**, possessing the C-6 hydroxy moiety on ring-A, were prepared starting from the commercially available 6-hydroxy-3-coumaranone in just one step employing acid- or base-catalyzed cross-aldol condensation with the appropriate benzaldehyde in analogy to reported procedure (Scheme 1) [18]. For sulfuretin analogs **1l**–**s**, which have the C-6 methoxy moiety on ring-A, 6-hydroxy-3-coumaranone was first methylated using iodomethane/potassium carbonate/DMF system to access yield 6-methoxy-3-coumaranone that was subjected to acid- or base-catalyzed cross-aldol condensation with the appropriate benzaldehyde to afford the desired compounds.

### 2.3. In Vitro Evaluation at 50 and 25 µM Concentrations 

To evaluate the direct antileishmanial activity of the prepared sulfuretin analogs **1a**–**s** as well as sulfuretin in comparison to the standard alkylphospholipid-based drug, erufosine, a model of *L. donovani* promastigotes, was used, and a resazurin-based assay [19,20] was recruited. The evaluation results at two single doses (50 and 25 µM) are summarized in Table 2.

**Figure 2:**
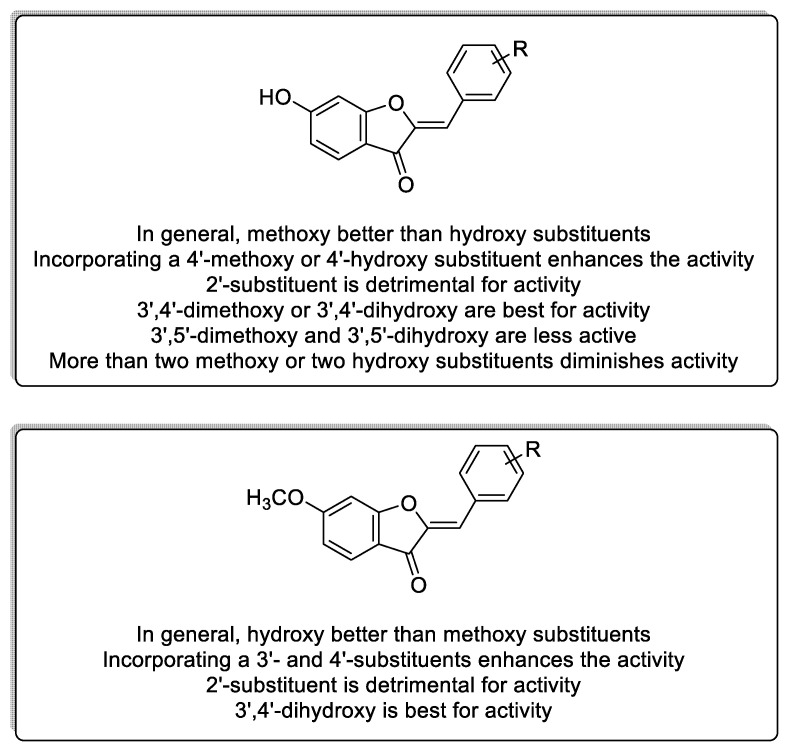
Summary of the revealed structure–antileishmanial activity relationship for evaluated sulfuretin analogs.

Analysis of the results unveiled an interesting activity profile for sulfuretin analogs. As shown in Table 2, sulfuretin, which possesses two vicinal hydroxy substituents at *meta* and *para* positions of ring-B coupled with C6-hydroxy moiety at ring-A, triggered almost 94% growth inhibition at 50 µM dose, but the inhibition decreased to nearly 54% at the 25 µM dose. Interestingly, displacement of the *para*-hydroxy moiety to the other *meta*-position of ring-B or adding a third hydroxy group to this other *meta*-position undermined the activity (compounds **1a** and **1b**, Table 2). Converting the highly polar hydrogen bond donor-acceptor monohydroxy group at ring-B to the less polar, bulkier and only hydrogen bond donor monomethoxy group resulted in compounds **1c**–**e** demonstrating weak activities even at the higher 50 µM dose. Despite their low activity, they provided information suggesting that the methoxy group at *para*-position of ring-B is (compound **1e**) is correlated with activity, while loss of activity, especially at the lower dose, is correlated with methoxy group at *ortho*-position of ring-B (compound **1c**). To explore the steric limits at *para*-position of ring B, compound **1f**, incorporating the more protruding methoxymethoxy moiety at *para*-position, was assessed. The resulted indicated the presence of a steric intolerance that resulted in lowering the activity. Combining two methoxy moieties or more at ring-B (analogs **1g**–**k**) provided more support to find position-dependent influences of methoxy moieties on activity. Thus, compound **1i**, having one methoxy group at *para*-position of ring-B and a second methoxy group at *meta*-position, showed high inhibition at both 50 and 25 µM doses (Table 2). Meanwhile, compounds which had one methoxy group at *ortho*-position of ring-B and a second methoxy group at *meta*-position in vicinal (compound **1g**) or distant relationship (compound **1h**) demonstrated very low activity even at the higher 50 µM dose. In comparison, compound **1j**, which had two methoxy groups at both *meta*-positions of ring-B, maintained good activity at both 50 and 25 µM doses, but possessed lower activity relative to the *meta* and *para*-dimethoxy substituted compound (**1i**). Nevertheless, compound **1k**, which combines three methoxy groups at all *meta*- and *para*-positions of ring-B, was a weak inhibitor, suggesting the presence of steric limits. Regarding library members that incorporate C6-methoxy moiety instead of the C6-hydroxy moiety at ring-A, the results again corroborated the influential role of substituents positions on ring-B. Thus, amongst compound **1l**–**n** possessing monohydroxy moieties at ring-B, compound **1n** incorporating monohydroxy moiety at *para*-position of ring-B was the most active compound, especially at the lower 25 µM dose. Meanwhile, derivative **1l**, which had monohydroxy moiety at *ortho*-position of ring-B, was the least active. Interestingly, compound **1m**, which had monohydroxy moiety at *meta*-position of ring-B, demonstrated significantly high activity at the high 50 µM dose, but high activity attrition was found at the lower 25 µM dose. Consistent with these findings, compound **1q**, having two hydroxy moieties at both *para*- and *meta*-positions of ring-B, was found as the most effective analogs among members **1o**–**q** combining dihydroxy moieties at ring-B with C6-methoxy moiety at ring A, while compound **1p**, with *ortho*- and *para*-dihydroxy moieties at ring-B, was completely inactive. However, significant high activity was triggered by compound **1o** having *ortho*- and *meta*-dihydroxy moieties at ring-B. The replacement of hydroxy groups at ring-B with methoxy groups in compounds which had C6-methoxy moiety at ring-A (compound **1r** and **1s**) did not yield significant improvement in activity. As a whole, the concluded structure–activity relationship might be summarized in Figure 2.

### 2.4. Cell Viability and Cytotoxicity Evaluation

An essential requirement in an antiparasitic agent is to elicit selective activity on the parasite while be safe on host cells. As sulfuretin and its analogs belong to the aurone class of compounds and some compounds of this class also can trigger the death of bone marrow-derived macrophages [15], the impact of sulfuretin analogs **1i** and **1q**, as well as the standard erufosine on the viability of host cells, was evaluated employing THP-1 cells, which are a human monocytic cell line that differentiates into macrophages. In addition, HEK293T cells, which are a variant of the human embryonic kidney cells, were used for viability evaluation. In contrast to the standard erufosine, which elicited a potential cytotoxicity and death of THP-1 cells at low micromolar concentration and a moderate cytotoxicity of HEK293T cells at nearly 28 µM concentration (Table 3), sulfuretin analog **1i** maintained good viability of THP-1 cells and excellent viability of HEK293T cells (CC_50_ near 100 and over 200 µM concentrations, respectively). Accordingly, sulfuretin analog **1i** might be anticipated to be a safe compound which is selective for the parasite rather than host cells. On the other side, sulfuretin analog **1q** showed significant cytotoxicity against both THP-1 and HEK293T cells. However, its result indicates a safer profile than erufosine against THP-1 cells and an equivalent cytotoxicity against HEK293T cells. 

### 2.5. Homology Modeling

**Figure 3:**
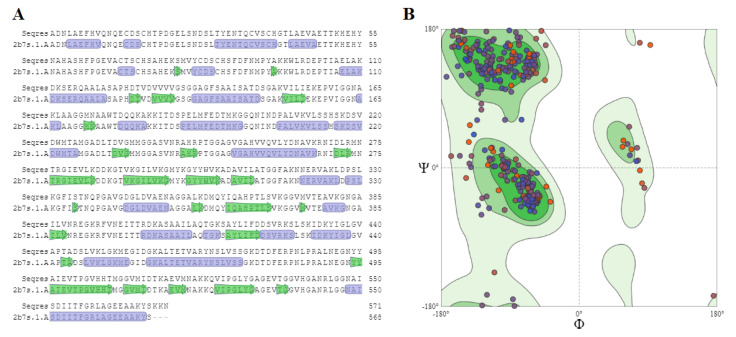
Sequence alignment and Ramachandran plot of the generated homology model of *L. donovani* NADH-dependent fumarate reductase: (**A**) Sequence alignment against chain A of S. frigidimarina fumarate reductase (PDB code: 2b7s) showing predicted secondary structures according to dictionary of secondary structure of protein (DSSP); (**B**) Ramachandran plot of φ/ψ of amino acid residues of the generated homology model indicated that most of them are located in the favored regions.

As there is an absence of resolved 3D coordinate files for *L. donovani* NADH-dependent fumarate reductase in the protein data bank, homology modeling was addressed to construct 3D coordinates for the structure of *L. donovani* NADH-dependent fumarate reductase employing the known amino acids sequence (Uniprot sequence ID: E9BRZ6). A template search with blast and HHblits using the target sequence of *L. donovani* NADH-dependent fumarate reductase has been conducted, as implemented in SWISS-MODEL [21,22]. In line with the known existence of two fumarate reductases: quinol-dependent fumarate reductase that incorporate FAD prosthetic group and, the other, NADH-dependent fumarate reductase that share the conserved catalytic region, the template search and alignment indicated that the flavoprotein subunit of S. frigidimarina fumarate reductase fumarate reductase (PDB code: 2b7s) was the best template covering all the sequence and possessing 36.75% identity. Using the aligned sequence (Figure 3A) and template coordinates, the homology model was generated with ProMod3 [23]. A Ramachandran plot of φ/ψ of amino acid residues of the built homology model showed that 90.52% of residues were in favored regions, while only 1.44% residues were in disfavored region (Figure 3B). This indicated acceptable quality and the model might be used in further studies. 

### 2.6. Molecular Docking

**Figure 4:**
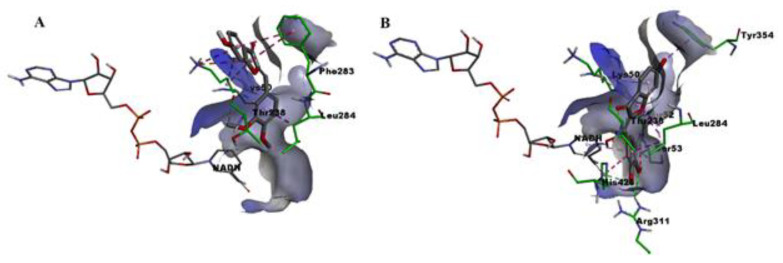
Predicted binding modes and interactions with *L. donovani* NADH-dependent fumarate reductase: (**A**) Predicted binding mode and interactions for sulfuretin analogs **1i**; (**B**) Predicted binding mode and interactions for sulfuretin analogs **1q**.

A docking study simulation was performed to obtain insights into the binding modes of sulfuretin analogs **1i** and **1q** to *L. donovani* NADH-dependent fumarate reductase after refinement with a brief dynamic simulation with the inhibitor sulfuretin within the fumarate site. As shown in Figure 4, analogs **1i** and **1q** could be docked successfully in the substrate binding pocket, demonstrating favorable binding energy scores between −5.44 and −6.36. The most favorable poses predicted as binding modes suggested that ring-B moieties of both compound analogs **1i** and **1q** were inserted in a relatively narrow pocket facing the NADH co-factor, while their benzofuranone moieties were docked in a wider pocket. However, analog **1q**, possessing a C6-methoxy moiety at ring-A, involved more intricated interactions, not only with amino acids of the binding pocket, but also involved hydrophobic π-alkyl interaction between ring-B and the 1,4-dihydronicotinamide moiety of the NADH co-factor (Figure 4B). In comparison, the inhibitor analogs **1i**, possessing C6-hydroxy moiety at ring-A, did not establish such interaction with NADH according to its most favorable pose (Figure 4A). Its docking position showed a slight upward shift so that the methoxy moiety at the *meta*-position of ring-B is involved in favorable interaction with Thr328 or Thr52, respectively. On the other hand, the dihydroxy groups at *meta*- and *ortho*-positions of ring-B of the most potent analog **1q** established four favorable hydrogen bonds with the three residues Ser53, Arg311 and His426. In addition, the C6-methoxy moiety at ring-A of analog **1q** established a favorable hydrophobic π-δ interaction with Tyr354. Meanwhile, Lys50, Thr52, Leu284, and Thr238 established a network of favorable interactions with the benzofuranone moiety, in addition to favorable interactions between ring-B of analog **1q** with Leu284 and His426. Meanwhile, analog **1i** in its slightly upward shifted docked position also established favorable interactions between the benzofuranone moiety and ring-B with Lys50 and Leu284, in addition to Thr238 in the case of analog **1i**. Collectively, it could be inferred that the substitution pattern decorating ring-B of analogs **1q** and **1i** are determinant for binding, while their benzofuranone moieties might enable it to establish a more favorable interaction network and, thus, might translate into good activity.

## 3. Discussion

The genus *Leishmania* involves a large number of pathogenic and non-pathogenic species. Around 20 *Leishmania* species are human-pathogens and the most fatal is *L. donovani* [24]. Yet, still there are unmet needs for discovery and development of effective and safe therapeutic agents for leishmaniasis. Motivated by the fact that multifunctional compounds targeting multiple pathways correlated with the disease might be more promising, sulfuretin analogs were proposed as multifunctional compounds against *L. donovani*, the deadliest leishmania species responsible for visceral leishmaniasis, via direct inhibiting growth and indirect via immunomodulation triggered from inhibition of the production of PGE_2_. Accordingly, repurposing a library of sulfuretin analogs capable of inhibiting the immunosuppressive PGE_2_ production by macrophages was attempted. Library members bearing diverse substituents oxygenation patterns were synthesized concisely in analogy to reports in the literature. Such known concise synthesis might be advantageous, as the cost of synthesis would be reflected in the total cost of the product. Thus, the economies of synthesis are an important factor, especially when it comes to development of a therapeutic agent for a neglected disease, such as leishmania, that afflicts mainly populations of low-income countries. In lieu of this, economies of the synthesis including step and atom economy [25] were considered whilst selecting such a library of sulfuretin analogs for evaluation. Considering the fact that *L. donovani* is the most fatal species, a model of *L. donovani* promastigotes was selected to evaluate the direct inhibition of growth of *L. donovani* promastigotes. The results indicated that *para*-position of ring-B is highly influential and also *meta*-position has a significant positive impact on activity, but to a lower extent relative to *para*-position. In addition, it unveiled that methoxy moieties at either ring-A or B coupled with maintenance hydroxy moieties of sulfuretin at the other ring affords the most potential hits. Amongst evaluated sulfuretin analogs, compounds **1i** retaining the C6-hydroxy moiety at ring-A, but possessing methoxy moieties in place of the polar dihydroxy moieties of sulfuretin ring-B, as well as analog **1q** retaining the polar dihydroxy moieties of sulfuretin ring-B, but incorporating a C6-methoxy moiety instead of the C6-hydroxy moiety at ring-A, were found as the most potential hit compounds. Cytotoxicity and safety evaluation of the promising analogs **1i** and **1q** demonstrated that analog **1i** could be a safe compound triggering the death of the parasite while possessing no to low cytotoxicity on employed tested host cells. However, analog **1q** elicited a significant cytotoxicity, but is still safer in comparison to erufosine. To get insights of the possible binding mode, an in silico study using a constructed homology model of *L. donovani* NADH-fumarate reductase as a molecular target of these compounds provided insights on the possible binding mode of these compounds. In lieu of the predicted importance of ring-B and its substituents, it might be essential for activity, while their benzofuranone moieties might be modulated to access more potential antileishmanial compounds. Overall, the accumulated results suggest sulfuretin analogs **1i** and **1q** as potential hit compound for further development of therapeutic agents against *L. donovani*.

## 4. Materials and Methods

### 4.1. Chemistry

All solvents and reagents have been purchased from commercial suppliers and used without any further purification. NMR spectra were acquired on Bruker Avance 400 spectrometer (400 MHz) or JEOL JNM-ECZ500R spectrometer (500 MHz). ^1^H NMR spectra were referenced to tetramethylsilane (*δ* = 0.00 ppm) as an internal standard. High resolution mass spectra (HRMS) were recorded on Jeol AccuTOF (JMS-T100TD) equipped with a DART (direct analysis in real time) ion source from ionsense, Tokyo, Japan in the positive modes. TLC was carried out using glass sheets pre-coated with silica gel 60F_254_ purchased by Merk and spots were visualized under UV lamp or using staining solutions, such as *p*-anisaldehyde solution, ninhydrin solution. 6-Methoxy-3-coumaranone and compounds **1a**–**1s** were in agreement with the reported literature [26] (Supplementary Materials).

#### General Method for Acid-Catalyzed Cross-Aldol Condensation to Prepare Sulfuretin Analogs

HCl was added dropwise to a solution of the appropriate 3-coumaranone derivative in methanol or ethanol. After complete dissolution, the appropriate benzaldehyde derivative was added dropwise. The mixture was heated at 60 °C for the specified time. After completion of the reaction (TLC), the reaction mixture was diluted with water, filtered and the collected precipitate was collected by filtration and dried under reduced vacuum before purification of the obtained crude products using column chromatography.

### 4.2. In Vitro Evaluation against L. donovani Promastigotes

Evaluation was conducted according to standard reported procedures [2] as described in supporting materials.

### 4.3. In Vitro Host Cells Viability and Cytotoxicity

Viabilities of host cells were evaluated via measuring the conversion of resazurin to resorufin. In brief, THP-1 or HEK293T cell (1000 cells per well) were seeded in 384-well plates. After seeding, the cells were exposed to tested compounds or standard drug for 3 days. Resazurin sodium salt (200 µM; R7017; Sigma-Aldrich, St. Louis, MO, USA) was then added, and incubated for a further 5 h. After fixation using 4% paraformaldehyde, the plates were analyzed using a Victor3TM plate reader (PerkinElmer, Inc., Waltham, MA, USA) at 590 nm (emission) and 530 nm (excitation).

### 4.4. Statistical Analysis

Results are expressed as the mean of triplicates. Significance of the results was confirmed relative to control, where *p* values ≤ 0.05 were regarded as statistically significant using one-way ANOVA followed by Tukey–Kramer as the post hoc test.

### 4.5. In Silico Simulation 

The amino acids sequence of *L. donovani* NADH-dependent fumarate reductase was retrieved from UniProt database (https://www.uniprot.org/uniprot/E9BRZ6; accessed on 25 August 2022). SWISS-MODEL was used for the construction of the homology model. The generated homology model was used for docking compounds **1i**, **1j** and **1q**. The results were visualized by DS Visualizer.

## Figures and Tables

**Table 1:**
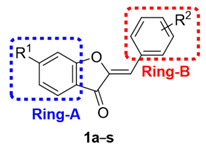
List of sulfuretin analogs (**1a**–**s**) and their known inhibition of PGE_2_ production triggered by sulfuretin and its analogs.

Comp.	R^1^	R^2^	PGE_2_ IC_50_ (µM) ^1^	Comp.	R^1^	R^2^	PGE_2_ IC_50_ (µM) ^1^
**1a**	6-Hydroxy	3′,5′-Dihydroxy	ND ^2^	**1k**	6-Hydroxy	2′,3′,4′-Trimethoxy	ND ^2^
**1b**	6-Hydroxy	3′,4′,5′-Trihydroxy	37.62	**1l**	6-Methoxy	2′-Hydroxy	2.22
**1c**	6-Hydroxy	2′-Methoxy	18.10	**1m**	6-Methoxy	3′-Hydroxy	13.15
**1d**	6-Hydroxy	3′-Methoxy	3.79	**1n**	6-Methoxy	4′-Hydroxy	35.82
**1e**	6-Hydroxy	4′-Methoxy	2.00	**1o**	6-Methoxy	2′,3′-Dihydroxy	ND ^2^
**1f**	6-Hydroxy	4′-Methoxymethoxy	ND ^2^	**1p**	6-Methoxy	2′,4′-Dihydroxy	8.80
**1g**	6-Hydroxy	2′,3′-Dimethoxy	ND ^2^	**1q**	6-Methoxy	3′,4′-Dihydroxy	4.90
**1h**	6-Hydroxy	2′,5′-Dimethoxy	ND ^2^	**1r**	6-Methoxy	2′-Methoxy	59.50
**1i**	6-Hydroxy	3′,4′-Dimethoxy	2.90	**1s**	6-Methoxy	3′-Methoxy	2.50
**1j**	6-Hydroxy	3′,5′-Dimethoxy	1.67	**Sulfuretin**	6-Hydroxy	3′,4′-Dihydroxy	5.90

^1^ Literature reported values [18]; **^2^** ND: not determined.

**Table 2:**
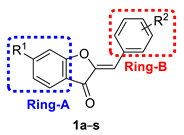
Antileishmanial activities of sulfuretin analogs (**1a**−**s**), sulfuretin and a standard alkylphospholipid-based drug.

Comp.	R^1^	R^2^	% Inhibition ± SD at 50 µM ^1^	% Inhibition ± SD at 25 µM ^1^
**1a**	6-Hydroxy	3′,5′-Dihydroxy	18.16 ± 2.32	12.14 ± 2.22
**1b**	6-Hydroxy	3′,4′,5′-Trihydroxy	36.74 ± 6.24	NI ^2^
**1c**	6-Hydroxy	2′-Methoxy	31.55 ± 3.76	1.06 ± 10.98
**1d**	6-Hydroxy	3′-Methoxy	29.19 ± 10.02	12.13 ± 0.60
**1e**	6-Hydroxy	4′-Methoxy	54.80 ± 1.21	43.34 ± 1.10
**1f**	6-Hydroxy	4′-Methoxymethoxy	29.34 ± 0.93	20.61 ± 3.96
**1g**	6-Hydroxy	2′,3′-Dimethoxy	25.37 ± 5.24	8.74 ± 1.05
**1h**	6-Hydroxy	2′,5′-Dimethoxy	19.05 ± 3.74	3.28 ± 1.31
**1i**	6-Hydroxy	3′,4′-Dimethoxy	**109.83 ± 0.37**	**106.54 ± 0.29**
**1j**	6-Hydroxy	3′,5′-Dimethoxy	**70.92 ± 1.19**	**78.10 ± 1.91**
**1k**	6-Hydroxy	2′,3′,4′-Trimethoxy	34.10 ± 0.37	13.58 ± 3.39
**1l**	6-Methoxy	2′-Hydroxy	27.02 ± 6.19	NI ^2^
**1m**	6-Methoxy	3′-Hydroxy	72.10 ± 1.49	35.47 ± 4.38
**1n**	6-Methoxy	4′-Hydroxy	63.95 ± 1.88	55.78 ± 1.48
**1o**	6-Methoxy	2′,3′-Dihydroxy	**83.55 ± 1.65**	**80.05 ± 1.32**
**1p**	6-Methoxy	2′,4′-Dihydroxy	NI ^2^	NI ^2^
**1q**	6-Methoxy	3′,4′-Dihydroxy	**108.11 ± 0.75**	**105.35 ± 0.13**
**1r**	6-Methoxy	2′-Methoxy	38.68 ± 0.67	20.45 ± 2.98
**1s**	6-Methoxy	3′-Methoxy	70.44 ± 0.56	59.23 ± 0.34
**Sulfuretin**	6-Hydroxy	3′,4′-Dihydroxy	93.71 ± 1.25	53.72 ± 0.57
**Erufosine**			107.63 ± 0.27	106.37 ± 0.97

^1^ % Inhibition of growth of *L. donovani* promastigotes after incubation with the specified concentration for 3 days relative to control; ^2^ NI: No inhibition.

**Table 3:**
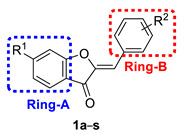
Cytotoxicity and cell viability evaluation for the most potential sulfuretin analogs (**1i** & **1q**) and the standard alkylphospholipid-based drug.

Comp.	R^1^	R^2^	CC_50_ (µM) ^1^	
			THP-1 Cell Line	HEK293T Cell Line
**1i**	6-Hydroxy	3′,4′-Dimethoxy	**96.86 ± 2.12**	**>200**
**1q**	6-Methoxy	3′,4′-Dihydroxy	15.08 ± 0.52	29.13 ± 0.98
**Erufosine**			1.91 ± 0.51	28.24 ± 3.87

^1^ CC_50_: The cytotoxic concentration of the employed compound (in micromolar concentration) that induces death of 50% of the cells relative to control.

## Data Availability

Data is contained within the article and supplementary material.

## Supplementary Materials

The following supporting information can be downloaded at: https://www.mdpi.com/article/10.3390/ph15091058/s1, Experimental procedures. References [18,27,28,29] are cited in the Supplementary Materials.
