# Supplementary material for: Design, Rational Repurposing, Synthesis, In Vitro Evaluation, Homology Modeling and In Silico Study of Sulfuretin Analogs as Potential Antileishmanial Hit Compounds
Source: Pharmaceuticals (Basel). 2022 Aug 26;15(9):1058. doi: 10.3390/ph15091058 (PMC9504330; doi:10.3390/ph15091058)
Supplement: Supplementary file 1 [file pharmaceuticals-15-01058-s001.zip › pharmaceuticals-1557490-supplementary.pdf]

Supporting information for

Design, rational repurposing, Synthesis, in vitro evaluation, homology modeling and in silico study of sulfuretin analogs as potential antileishmanial hit compounds

Ahmed H.E. Hassan[1,2], Trong-Nhat Phan[3], Suyeon Moon[4], Yeonwoo Choi[4], Joo Hwan No[3], Yong Sup Lee[2,5,*]

[1] *Department of Medicinal Chemistry, Faculty of Pharmacy, Mansoura University, Mansoura 35516, Egypt.*

[2] *Medicinal Chemistry Laboratory, Department of Pharmacy, College of Pharmacy, Kyung Hee University, Seoul 02447, Republic of Korea.*

[3] *Host-Parasite Research Laboratory, Institut Pasteur Korea, Seongnam-si, Gyeonggi-do 13488, Republic of Korea.*

[4] *Department of Fundamental Pharmaceutical Sciences, Kyung Hee University, Seoul 02447, Republic of Korea.*

[5] *Department of Life and Nanopharmaceutical Sciences, Kyung Hee University, Seoul 02447, Republic of Korea.*

[*]**Corresponding authors:** Yong Sup Lee (e-mail: kyslee@khu.ac.kr)

1. Chemistry

1.1. Preparation of 6-methoxy-3-coumaranone [1]

Methyl iodide (1.4 mL) was added to a mixture of 6-hydroxy-3-coumaranone (2.0 g, 13 mmol), anhydrous potassium carbonate (3.6 g, 22.1 mmol) in DMF (30 mL). The mixture was stirred overnight at rt. After quenching the reaction with cold water (60 mL), it was stored a refrigerator for 23 h. The solid product was collected by filtration and dried. Crystallization from cyclohexane afforded the above titled compound (1.2g. 58% yield).

^{1}H NMR (400 MHz, $CDCl_3$): *δ* 7.58 (d, *J* = 8.0 Hz, 1H), 6.66 (dd, *J* = 8.0, 2.0 Hz, 1H), 6.55 (d, *J* = 2.0 Hz, 1H), 4.63 (s, 2H), 3.88 (s, 3H, OCH_3).

1.2. Preparation of 4-(methoxymethoxy)benzaldehyde [2]

To a solution of 4-hydroxy benzaldehyde (2.5 g, 20.0 mmol) in dichloromethane (30 ml), *N*,*N*-diisopropylethylamine (3.5 mL, 20.0 mmol) was added followed by dropwise addition of chloromethyl methyl ether (1.5 ml, 20.0 mmol) at 0 °C. After stirring at room temperature for 6 hours, the reaction was quenched with water, extracted with dichloromethane and the combined organic extracts were dried over anhydrous $MgSO_4$. Evaporation under reduced pressure afforded oily crude product which was purified by column chromatography to afford the above titled compounds (2.9 g, 78% yield).

^{1}H NMR (400 MHz, $CDCl_3$) δ 9.91 (s, 1H), 7.89 (tt, *J* = 5.2, 2.6, 1.8 Hz, 2H), 7.22 (tt, *J* = 6.8, 2.8, 1.6 Hz, 2H), 5.78 (s, 2H), 3.42 (s, 3H).

(*Z*)-2-(3,5-Dihydroxybenzylidene)-6-hydroxybenzofuran-3(2*H*)-one (1a)

Compound 1a was obtained according to general procedure 4.1.1 using 6-hydroxy-3-coumaranone (144.8 mg, 1.0 mmol), ethanol (30 mL), HCl (12N, 3 mL) and 3,5-dihydroxybenzaldehyde (133.3

mg, 1.0 mmol). Reaction time was 6 hours and it was purified by column chromatography (silica gel, EtOAc/*n*-hexane = 1:2) to afford the above titled compound 1a (25.3 mg, 7% yield).

^{1}H NMR (500 MHz, DMSO-d_6): δ 11.2 (brs, 1H), 9.47 (s, 2H), 7.59 (d, J = 8.4 Hz, 1H), 6.78 (d, J = 2.0 Hz, 2H), 6.68 (m, 2H), 6.52 (s, 1H), 6.27 (t, J = 2.2 Hz, 1H); ^{13}C NMR (125 MHz, DMSO-d_6): δ 182.0, 168.4, 167.1, 159.1, 147.7, 133.8, 126.6, 113.6, 113.3, 111.6, 109.8, 105.0, 98.9; HRMS calcd for $C_{15}H_{11}O_5$ $[M+H]^+$ 271.0607, found 271.0623.

(*Z*)-6-Hydroxy-2-(3,4,5-trihydroxybenzylidene)benzofuran-3(2*H*)-one (1b) [3]

Compound **1b** was obtained according to general procedure 4.1.1 using 6-hydroxy-3-coumaranone (300.0 mg, 2.0 mmol), ethanol (30 mL), HCl (12N, 3 mL) and 3,4,5-trihydroxybenzaldehyde (345.4 mg, 2.0 mmol). Reaction time was 6 hours and it was purified by column chromatography (silica gel, EtOAc/*n*-hexane = 1:2) to afford the above titled compound **1b** (412.0 mg, 72% yield). ^{1}H NMR (400 MHz, DMSO-d_6): δ 11.15 (brs, 1H), 9.18 (brm, 2H), 7.60 (d, J = 8.4 Hz, 1H), 6.95 (s, 2H), 6.73–6.69 (m, 2H), 6.54 (s, 1H); ^{13}C NMR (100 MHz, DMSO-d_6): δ 181.5, 167.8, 166.5, 146.5, 146.1, 136.7, 126.2, 122.6, 113.6, 113.3, 111.3, 98.7.

(*Z*)-6-Hydroxy-2-(2-methoxybenzylidene)benzofuran-3(2*H*)-one (1c) [3]

Compound **1c** was obtained according to general procedure 4.1.1 using 6-hydroxy-3-coumaranone (300.6 mg, 2.0 mmol), ethanol (30 mL), HCl (12N, 3 mL) and 2-methoxybenzaldehyde (0.215 mL, 2.0 mmol). Reaction time was 9 hours and it was purified by column chromatography (silica gel, dichloromethane/methanol = 15:1) to afford the above titled compound **1c** (419.3 mg, 78% yield). ^{1}H NMR (400 MHz, DMSO-d_6): δ 11.19 (brs, 1H), 8.15 (dd, J = 6.3, 1.4 Hz, 1H), 7.62 (d, J = 8.4 Hz, 1H), 7.43 (m, 1H), 7.10 (m, 2H), 7.06 (s, 1H), 6.79 (d, J = 1.84 Hz, 1H), 6.72 (dd, J = 6.5, 1.8 Hz, 1H), 3.89 (s, 3H); ^{13}C NMR (100 MHz, DMSO-d_6): δ 181.3, 167.8, 166.4, 158.0, 147.3, 131.4,

130.9, 125.9, 120.8, 120.2, 113.0, 112.7, 111.4, 103.6, 98.6, 55.7.

(*Z*)-6-Hydroxy-2-(3-methoxybenzylidene)benzofuran-3(2*H*)-one (1d) [3]

Compound **1d** was obtained according to general procedure 4.1.1 using 6-hydroxy-3-coumaranone (301.2 mg, 2.0 mmol), ethanol (30 mL), HCl (12N, 3 mL) and 3-methoxybenzaldehyde (0.245 mL, 2.0 mmol). Reaction time was 9 hours and it was purified by column chromatography (silica gel, dichloromethane/methanol = 15:1) to afford the above titled compound **1d** (493.3 mg, 92% yield). ^{1}H NMR (400 MHz, DMSO-d_6): δ 11.16 (brs, 1H), 7.61 (d, J = 8.4 Hz, 1H), 7.52 (d, J = 7.7 Hz, 1H), 7.47 (d, J = 2.1 Hz, 1H), 7.39 (t, J = 8.0 Hz, 1H), 6.99 (dd, J = 5.6, 2.5, 1.9 Hz, 1H), 6.79 (d, J = 1.8 Hz, 1H), 6.74 (s, 1H), 6.71 (dd, J = 6.4, 1.9 Hz, 1H), 3.80 (s, 3H); ^{13}C NMR (100 MHz, DMSO-d_6): δ 181.9, 168.4, 167.1, 159.8, 147.9, 133.7, 130.4, 126.4, 123.9, 116.7, 115.8, 113.6, 113.1, 110.7, 99.1, 55.6

(*Z*)-6-Hydroxy-2-(4-methoxybenzylidene)benzofuran-3(2*H*)-one (1e) [3]

Compound **1e** was obtained according to general procedure 4.1.1 using 6-hydroxy-3-coumaranone (301.2 mg, 2.0 mmol), ethanol (30 mL), HCl (12N, 3 mL) and 4-methoxybenzaldehyde (0.250 mL, 2.0 mmol). Reaction time was 9 hours and it was purified by column chromatography (silica gel, dichloromethane/methanol = 15:1) to afford the above titled compound **1e** (467.0 mg, 87% yield). ^{1}H NMR (400 MHz, DMSO-d_6): δ 11.17 (brs, 1H), 7.98 (d, J = 8.8 Hz, 2H), 7.68 (d, J = 8.4 Hz, 1H), 7.12 (d, J = 8.8 Hz, 2H), 6.86 (d, J = 1.8 Hz, 1H), 6.84 (s, 1H), 6.79 (dd, J = 6.5, 1.9 Hz, 1H), 3.89 (s, 3H); ^{13}C NMR (100 MHz, DMSO-d_6): δ 181.7, 168.1, 166.7, 160.9, 146.6, 133.4, 126.2, 125.0, 115.0, 113.5, 113.3, 111.2, 99.0, 55.7.

(*Z*)-6-Hydroxy-2-(4-(methoxymethoxy)benzylidene)benzofuran-3(2*H*)-one (1f) [4]

Compound **1f** was obtained according to general procedure 4.1.2 using 6-hydroxy-3-coumaranone (905.0 mg, 9.0 mmol), ethanol (30 mL), KOH (50%, 4.5 mL) and 4-(methoxymethoxy)benzaldehyde (972.2 mg, 6.0 mmol). Reaction time was 6 hours and it was purified by column chromatography (silica gel, EtOAc/*n*-hexane = 1:3) to afford the above titled compound **1f** (1540.7 mg, 86% yield).

^{1}H NMR (400 MHz, DMSO-d_6) δ 11.16 (brs, 1H), 7.92 (d, J = 8.8 Hz, 2H), 7.62 (d, J = 8.4 Hz, 1H), 7.14 (d, J = 8.8 Hz, 2H), 6.79 (d, J = 1.8 Hz, 2H), 6.71 (dd, J = 8.4, 1.8 Hz, 1H), 5.27 (s, 2H), 3.37 (s, 3H) ; ^{13}C NMR (125 MHz, DMSO-d_6): δ 181.9, 168.3, 166.9, 158.4, 146.9, 133.4, 133.2, 126.4, 126.1, 117.0, 116.3, 113.5, 111.1, 99.1, 98.3, 94.2, 56.3.

(*Z*)-2-(2,3-Dimethoxybenzylidene)-6-hydroxybenzofuran-3(2*H*)-one (1g) [4]

Compound **1g** was obtained according to general procedure 4.1.1 using 6-hydroxy-3-coumaranone (300 mg, 2.0 mmol), methanol (10 mL), HCl (12N, 3 mL) and 2,3-dimethoxybenzaldehyde (398 mg, 2.4 mmol). Reaction time was 2 hours and it was purified by column chromatography (silica gel, dichloromethane/methanol = 15:1) to afford the above titled compound **1g** (578 mg, 97% yield).

^{1}H NMR (400 MHz, DMSO-d_6): δ δ 11.12 (brs, 1H), 7.80 (dd, J = 8.0, 1.6 Hz, 1H), 7.65 (d, J = 8.4 Hz, 1H), 7.24 (t, J = 8.0 Hz, 1H), 7.17 (dd, J = 8.0, 1.6 Hz, 1H), 6.98 (s, 1H), 6.79 (d, J = 1.8 Hz, 1H), 6.74 (dd, J = 8.4, 1.8 Hz, 1H), 3.86 (s, 3H), 3.83 (s, 3H); ^{13}C NMR (125 MHz, DMSO-d_6): δ 182.0, 168.5, 167.2, 153.1, 152.7, 148.7, 148.5, 126.6, 125.1, 122.9, 115.1, 113.7, 113.2, 104.2, 99.2, 61.6, 56.3.

(*Z*)-2-(2,5-Dimethoxybenzylidene)-6-hydroxybenzofuran-3(2*H*)-one (1h) [4]

Compound **1h** was obtained according to general procedure 4.1.1 using 6-hydroxy-3-coumaranone (300 mg, 2.0 mmol), ethanol (10 mL), HCl (12N, 3 mL) and 2,5-dimethoxybenzaldehyde (398 mg, 2.4 mmol). Reaction time was 3 hours and it was purified by column chromatography (silica gel, dichloromethane/methanol = 15:1) to afford the above titled compound **1h** (553 mg, 93% yield).

^{1}H NMR (400 MHz, DMSO-d_6): δ 11.2 (brs, 1H), 7.70 (d, J = 2.4 Hz, 1H), 7.64 (d, J = 8.4 Hz, 1H), 7.05-7.02 (m, 3H), 6.82 (d, J = 1.8 Hz, 1H), 6.74 (dd, J = 8.4, 1.8 Hz, 1H), 3.85 (s, 3H), 3.79 (s, 3H); ^{13}C NMR (100 MHz, DMSO-d_6): δ 181.9, 168.4, 167.1, 153.6, 153.1, 148.0, 126.5, 121.4, 117.0, 116.7, 113.6, 113.3, 113.0, 104.1, 99.3, 56.7, 56.0.

(*Z*)-2-(3,4-Dimethoxybenzylidene)-6-hydroxybenzofuran-3(2*H*)-one (1i) [3]

Compound **1i** was obtained according to general procedure 4.1.1 using 6-hydroxy-3-coumaranone (300.3 mg, 2.0 mmol), ethanol (10 mL), HCl (12N, 3 mL) and 3,4-dimethoxybenzaldehyde (333.1 mg, 2.0 mmol). Reaction time was 9 hours and it was purified by column chromatography (silica gel, dichloromethane/methanol = 20:1) to afford the above titled compound **1i** (300.1 mg, 50% yield).

^{1}H NMR (400 MHz, DMSO-d_6): δ 11.1 (brs, 1H), 7.62-7.54 (m, 3H), 7.06 (d, J = 8.2 Hz, 1H), 6.80 (d, J = 15.0 Hz, 1H), 6.77 (s, 1H), 6.71 (d, J = 8.3 Hz, 1H), 3.82 (s, 6H); ^{13}C NMR (100 MHz, DMSO-d_6): δ 181.6, 168.0, 166.7, 150.8, 149.1, 146.6, 126.2, 125.5, 125.2, 114.6, 113.4, 113.0, 112.3, 111.6, 99.0, 55.9 (2C).

(*Z*)-2-(3,5-Dimethoxybenzylidene)-6-hydroxybenzofuran-3(2*H*)-one (1j) [3]

Compound **1j** was obtained according to general procedure 4.1.1 using 6-hydroxy-3-coumaranone (300.6 mg, 2.0 mmol), ethanol (30 mL), HCl (12N, 3 mL) and 3,5-dimethoxybenzaldehyde (333.4 mg, 2.0 mmol). Reaction time was 9 hours and it was purified by column chromatography (silica

gel, dichloromethane/methanol = 15:1) to afford the above titled compound **1j** (403.0 mg, 67% yield).

^{1}H NMR (400 MHz, Pyridine-d_5): δ 7.82 (d, J = 8.4 Hz, 1H), 7.29 (d, J = 2.1 Hz, 2H), 7.19 (s, 1H), 7.02 (dd, J = 5.2, 1.8 Hz, 1H), 6.93 (dd, J = 6.5, 1.8 Hz, 1H), 6.70 (dd, J = 2.1 Hz, 1H), 3.72 (s, 6H).

(*Z*)-6-Hydroxy-2-(2,3,4-trimethoxybenzylidene)benzofuran-3(2*H*)-one (1k) [4]

Compound **1k** was obtained according to general procedure 4.1.1 using 6-hydroxy-3-coumaranone (300 mg, 2.0 mmol), ethanol (10 mL), HCl (12N, 3 mL) and 2,3,4-trimethoxybenzaldehyde (470 mg, 2.4 mmol). Reaction time was 4 hours and the crude product was purified by column chromatography (silica gel, dichloromethane/methanol = 15:1) to afford the above titled compound **1k** (628 mg, 95% yield).

^{1}H NMR (500 MHz, DMSO-d_6) δ 11.16 (brs, 1H), 7.90 (d, J = 8.4 Hz, 1H), 7.57 (d, J = 8.1 Hz, 1H), 6.96 (d, J = 6.9 Hz, 1H), 6.86 (s, 1H), 6.73 (d, J = 1.9 Hz, 1H), 6.67 (dd, J = 8.1, 1.9 Hz, 1H), 3.84 (s, 6H), 3.74 (s, 3H); ^{13}C NMR (125 MHz, DMSO-d_6): δ 181.6, 168.2, 166.9, 155.7, 153.5, 147.4, 142.2, 126.9, 126.5, 118.8, 113.5 (2C)p, 109.2, 104.6, 99.1, 62.2, 61.0, 56.6.

(*Z*)-2-(2-Hydroxybenzylidene)-6-methoxybenzofuran-3(2*H*)-one (1l) [3]:

Compound **1l** was obtained according to general procedure 4.1.1 using 6-methoxy-3-coumaranone (328 mg, 2.0 mmol), ethanol (10 mL), HCl (12N, 3 mL) and 2-hydroxybenzaldehyde (0.21 mL, 2.0 mmol). Reaction time was 12 hours and the crude product was purified by column chromatography (silica gel, dichloromethane/methanol = 15:1) to afford the above titled compound **1l** (408 mg, 76% yield).

^{1}H NMR (400 MHz, DMSO-d_6): δ 10.4 (s, 1H), 8.15 (dd, J = 8.0, 1.2 Hz, 1H), 7.70 (d, J = 8.4 Hz,

1H), 7.31 (td, J = 8.0, 1.2 Hz, 1H), 7.17 (d, J = 2.0 Hz, 1H), 7.16 (s, 1H), 6.98–6.93 (m, 2H), 6.87 (dd, J = 8.4, 2.0 Hz, 1H), 3.93 (s, 3H); ^{13}C NMR (100 MHz, DMSO-d_6): δ 181.6, 167.8, 167.2, 157.2, 146.8, 131.5, 131.0, 125.4, 119.6, 118.7, 115.8, 114.0, 112.6, 105.2, 97.1, 56.4;

(*Z*)-2-(3-Hydroxybenzylidene)-6-methoxybenzofuran-3(2*H*)-one (1m) [3]

Compound **1m** was obtained according to general procedure 4.1.1 using 6-methoxy-3-coumaranone (328 mg, 2.0 mmol), ethanol (5 mL), HCl (12N, 3 mL) and 3-hydroxybenzaldehyde (184 mg, 1.5 mmol). Reaction time was 14 hours and the crude product was purified by column chromatography (silica gel, dichloromethane/methanol = 15:1) to afford the above titled compound **1m** (147 mg, 27% yield).

^{1}H NMR (400 MHz, DMSO-d_6): δ 9.73 (s, 1H), 7.72 (d, J = 8.4 Hz, 1H), 7.41–7.40 (m, 2H) 7.32 (t, J = 8.0 Hz, 1H), 7.15 (d, J = 2.0, 1H), 6.88–6.86 (m, 2H), 6.76 (s, 1H), 3.94 (s, 3H); ^{13}C NMR (100 MHz, DMSO-d_6): δ 182.2, 168.5, 167.9, 158.1, 147.6, 133.5, 130.4, 126.0, 122.9, 118.0, 117.7, 114.3, 113.2, 111.7, 97.6, 56.9.

(*Z*)-2-(4-Hydroxybenzylidene)-6-methoxybenzofuran-3(2*H*)-one (1n) [3]

Compound **1n** was obtained according to general procedure 4.1.1 using 6-methoxy-3-coumaranone (328 mg, 2.0 mmol), ethanol (15 mL), HCl (12N, 3 mL) and 4-hydroxybenzaldehyde (244 mg, 2.0 mmol). Reaction time was 5 hours and the crude product was purified by column chromatography (silica gel, dichloromethane/methanol = 15:1) to afford the above titled compound **1n** (222 mg, 39% yield).

^{1}H NMR (400 MHz, DMSO-d_6): δ 10.2 (brs, 1H), 7.87 (d, J = 8.8 Hz, 2H), 7.69 (d, J = 8.4 Hz, 1H), 7.16 (d, J = 2.0 Hz, 1H), 6.91 (d, J = 8.8 Hz, 2H), 6.87 (dd, J = 8.4, 2.0 Hz, 1H), 6.80 (s, 1H), 3.93 (s, 3H); ^{13}C NMR (100 MHz, DMSO-d_6): δ 181.4, 167.5, 167.0, 159.4, 145.6, 133.4 (2C), 125.2, 122.9, 116.0 (2C), 114.2, 112.4, 112.0, 97.0, 56.3.

(*Z*)-2-(2,3-Dihydroxybenzylidene)-6-methoxybenzofuran-3(2*H*)-one (1o)

Compound **1o** was obtained according to general procedure 4.1.1 using 6-methoxy-3-coumaranone (328.9 mg, 2.0 mmol), ethanol (10 mL), HCl (12N, 3 mL) and 2,3-dihydroxybenzaldehyde (277.7 mg, 2.0 mmol). Reaction time was 8 hours and the crude product was purified by column chromatography (silica gel, EtOAc/*n*-hexane = 1:2) to afford the above titled compound **1o** (147.1 mg, 26% yield). ^{1}H NMR (500 MHz, DMSO-d_6): δ 7.64 (d, J = 8.6 Hz, 1H), 7.57 (dd, J = 8.0, 1.5 Hz, 1H), 7.14 (s, 1H), 7.11 (d, J = 2.2 Hz, 1H), 6.82–6.79 (m, 2H), 6.71 (t, J = 7.9 Hz, 1H), 3.88 (s, 3H) , 3.12 (s, 1H), , 2.49 (s, 1H); ^{13}C NMR (125 MHz, DMSO-d_6): δ 182.1, 168.3, 167.7, 147.2, 146.8, 146.1, 125.9, 121.8, 119.9, 119.8, 117.4, 114.6, 113.1, 106.5, 97.7, 40.9; HRMS calcd for $C_{16}H_{13}O_5$ $[M+H]^+$ 285.0763, found 285.0764.

(*Z*)-2-(2,4-Dihydroxybenzylidene)-6-methoxybenzofuran-3(2*H*)-one (1p) [3]

Compound **1p** was obtained according to general procedure 4.1.1 using 6-methoxy-3-coumaranone (329 mg, 2.0 mmol), ethanol (14 mL), HCl (12N, 3 mL) and 2,4-hydroxybenzaldehyde (277 mg, 2.0 mmol). Reaction time was 10 hours and the crude product was purified by column chromatography (silica gel, dichloromethane/methanol = 15:1) to afford the above titled compound **1p** (71 mg, 12% yield).

^{1}H NMR (400 MHz, DMSO-d_6): δ 10.40 (s, 1H), 10.10 (s, 1H), 8.02 (d, J = 8.4 Hz, 1H), 7.67 (d, J = 8.8 Hz, 1H), 7.14 (d, J = 2.4 Hz, 1H), 7.10 (s, 1H), 6.86 (dd, J = 8.8, 2.4 Hz, 1H), 6.42–6.38 (m, 2H), 3.90 (s, 3H); ^{13}C NMR (100 MHz, DMSO-d_6): δ 181.1, 167.2, 166.7, 161.0, 159.3, 145.0, 132.5, 125.0, 114.5, 112.3, 110.5, 108.3, 106.5, 102.3, 97.0, 56.3.

(*Z*)-2-(3,4-Dihydroxybenzylidene)-6-methoxybenzofuran-3(2*H*)-one (1q) [3]

Compound **1q** was obtained according to general procedure 4.1.1 using 6-methoxy-3-coumaranone (329 mg, 2.0 mmol), ethanol (7 mL), HCl (12N, 3 mL) and 3,4-

hydroxybenzaldehyde (273 mg, 2.0 mmol). Reaction time was 4 hours and the crude product was purified by column chromatography (silica gel, dichloromethane/methanol = 15:1) to afford the above titled compound **1q** (327 mg, 58% yield).

^{1}H NMR (400 MHz, methanol-d_4) δ 7.68 (dd, J = 8.8, 4.4 Hz, 1H), 7.52 (s, 1H), 7.29 (dd, J = 8.4, 2.4 Hz, 1H), 6.95 (d, J = 2.4 Hz, 1H), 6.86–6.82 (m, 2H), 6.73 (d, J = 4.4 Hz, 1H), 3.96 (s, 3H); ^{13}C NMR (100 MHz, DMSO-d_6) δ 181.3, 167.4, 166.9 (2C), 148.2, 145.5, 125.2, 124.6, 123.3, 118.2, 116.0, 114.3, 112.5, 112.4, 96.9, 56.3.

(*Z*)-6-Methoxy-2-(2-methoxybenzylidene)benzofuran-3(2*H*)-one (1r) [3]:

Compound **1r** was obtained according to general procedure 4.1.1 using 6-methoxy-3-coumaranone (329 mg, 2.0 mmol), ethanol (5 mL), HCl (12N, 3 mL) and 2-methoxybenzaldehyde (288 mg, 2.1 mmol). Reaction time was 5 hours and the crude product was purified by column chromatography (silica gel, dichloromethane/methanol = 15:1) to afford the above titled compound **1r** (387 mg, 69% yield).

^{1}H NMR (400 MHz, $CDCl_3$) δ 8.27 (dd, J = 8.0, 2.0 Hz, 1H), 7.72 (d, J = 8.4 Hz, 1H), 7.40 (s, 1H), 7.38 (td, J = 8.0, 2.0 Hz, 1H), 7.07 (t, J = 8.0 Hz, 1H), 6.94 (d, J = 8.0 Hz, 1H), 6.77–6.73 (m, 2H), 3.92 (s, 3H), 3.90 (s, 3H); ^{13}C NMR (100 MHz, $CDCl_3$) δ 183.0, 168.4, 167.2, 158.7, 147.9, 131.8, 131.1, 125.8, 121.5, 120.8, 115.1, 112.0, 110.8, 106.1, 96.6, 56.0, 55.6.

(*Z*)-6-Methoxy-2-(3-methoxybenzylidene)benzofuran-3(2*H*)-one (1s) [3]:

Compound **1s** was obtained according to general procedure 4.1.1 using 6-methoxy-3-coumaranone (329 mg, 2.0 mmol), ethanol (5 mL), HCl (12N, 3 mL) and 3-methoxybenzaldehyde (0.24 mL, 2.0 mmol). Reaction time was 17 hours and the crude product was purified by column chromatography (silica gel, dichloromethane/methanol = 15:1) to afford the above titled compound **1s** (200 mg, 36% yield).

^{1}H NMR (400 MHz, $CDCl_3$) δ 7.72 (d, J = 8.0 Hz, 1H), 7.48–7.46 (m, 2H), 7.38 (t, J = 8.0 Hz, 1H), 6.96 (m, J = 8.0, 2.0 Hz, 1H), 6.79–6.75 (m, 3H), 3.93 (s, 3H), 3.88 (s, 3H); ^{13}C NMR (100 MHz, $CDCl_3$) δ 183.0, 168.6, 167.5, 159.8, 148.0, 133.7, 129.8, 125.9, 124.1, 116.5, 115.4, 114.9, 112.2, 111.7, 96.7, 56.1, 55.4.

2. *In vitro* biological evaluations using *L. donovani* promastigotes

2.1. Cell culture of parasite

L. donovani MHOM/SD/62/1S-CL2D parasites were cultured as promastigotes at 28 °C in M199 medium (Sigma-Aldrich, St. Louis, MO, USA) with 40 mM HEPES, 0.1 mM adenine, 0.0001% biotin, and 4.62 mM $NaHCO_3$ supplemented with 10% fetal bovine serum (FBS, Gibco, Carlsbad, CA, USA), 100 µ/mL penicillin (Gibco), and 100 µg/mL streptomycin (Gibco). Parasites were sub-cultured every 3 or 4 days and maintained for 10 passages.

2.2. Assay of parasite growth inhibition

The values of growth inhibition of *L. donovani* promastigotes were determined based on metabolism of resazurin to resorufin by aerobic respiration of metabolically active cells using 384-well plates that were seeded with *L. donovani* promastigotes (5×10^4 cells per well) and incubated with tested compounds for 3 days followed by addition of Resazurin sodium salt (200 µM; R7017; Sigma-Aldrich, St. Louis, MO, USA) and further incubation for 5 hours then the cells were fixed (4% paraformaldehyde). The plates were analyzed using a Victor3TM plate reader (PerkinElmer, Inc., Waltham, MA, USA) at 590 nm (emission) and 530 nm (excitation). Erufosine was used as a reference standard. All measured and calculated values are the average of triplicates.

References

[1] K.Z. Łączkowski, M.M. Pakulski, M.P. Krzemiński, P. Jaisankar, M. Zaidlewicz, Asymmetric synthesis of N-substituted N-hydroxyureas, Tetrahedron: Asymmetry, 19 (2008) 788-795.

[2] N. Stuhr-Hansen, J.K. Sørensen, K. Moth-Poulsen, J.B. Christensen, T. Bjørnholm, M.B. Nielsen, Synthetic protocols and building blocks for molecular electronics, Tetrahedron, 61 (2005) 12288-12295.
[3] S.Y. Shin, M.C. Shin, J.S. Shin, K.T. Lee, Y.S. Lee, Synthesis of aurones and their inhibitory effects on nitric oxide and PGE2 productions in LPS-induced RAW 264.7 cells, Bioorg. Med. Chem. Lett., 21 (2011) 4520-4523.
[4] Y.H. Lee, M.C. Shin, Y.D. Yun, S.Y. Shin, J.M. Kim, J.M. Seo, N.-J. Kim, J.H. Ryu, Y.S. Lee, Synthesis of aminoalkyl-substituted aurone derivatives as acetylcholinesterase inhibitors, Bioorg. Med. Chem., 23 (2015) 231-240.
